# Definitive Surgical Treatment for Cholelithiasis in Selected Patients with Liver Cirrhosis

**DOI:** 10.1155/1995/59456

**Published:** 1995

**Authors:** Cheng-Chung Wu, Chi-Jou Hwang, June-Hei Fang, Tse-Jia Liu

**Affiliations:** Division of General Surgery Department of Surgery Taichung Veterans General Hospital Taiwan

## Abstract

To determine whether definitive surgery such as cholecysectomy or extraction of bile duct stones is appropriate in cirrhotic patients the results of definitive surgery have been reviewed retrospectively in a group of 112 cirrhotic patients with cholelithiasis. Eighty-seven of these patients underwent definitive surgery for gallstones and the remaining 25 were treated conservatively.

Child's criteria were applied to each patient. Patients with Child's grade A disease had fewer emergency procedures, operative blood loss and transfusion were less and they had a shorter hospital stay compared with patients with grades B and C. There were 4 deaths after definitive surgery for emergency conditions and these were all in Child's grade C. Of the 83 survivors after definitive procedures .78 patients (93.9%) were still alive 52.8 months later without any biliary tract symptoms. Of the 25 patients undergoing conservative treatment 2 were Child's B and 23 were Child's C grade.

We suggest that definitive surgery can be carried out safely, in Child's A and B cirrhotic patients, either electively or as an emergency. However, a more conservative approach is advisable in Child C patients with acute conditions and definitive surgery is recommended as an elective procedure after the liver function has improved.


*HPB Surgery*, 1995, Vol. 9, pp. 43–46
Reprints available directly from the publisher
Photocopying permitted by license only

© 1995 OPA (Overseas Publishers Association)
Amsterdam B.V. Published in The Netherlands
by Harwood Academic Publishers GmbH
Printed in Malaysia

### Definitive Surgical Treatment for Cholelithiasis in Selected Patients with Liver Cirrhosis

CHENG-CHUNG WU, CHI-JOU HWANG, JUNE-HEI FANG, and TSE-JIA LIU

Division of General Surgery, Department of Surgery, Taichung Veterans General Hospital, Taiwan, R.O.C.

To determine whether definitive surgery such as cholecysectomy or extraction of bile duct stones is appropriate in cirrhotic patients the results of definitive surgery have been reviewed retrospectively in a group of 112 cirrhotic patients with cholelithiasis. Eighty-seven of these patients underwent definitive surgery for gallstones and the remaining 25 were treated conservatively.

Child's criteria were applied to each patient. Patients with Child's grade A disease had fewer emergency procedures, operative blood loss and transfusion were less and they had a shorter hospital stay compared with patients with grades B and C. There were 4 deaths after definitive surgery for emergency conditions and these were all in Child's grade C. Of the 83 survivors after definitive procedures 78 patients (93.9%) were still alive 52.8 months later without any biliary tract symptoms. Of the 25 patients undergoing conservative treatment 2 were Child's B and 23 were Child's C grade.

We suggest that definitive surgery can be carried out safely, in Child's A and B cirrhotic patients, either electively or as an emergency. However, a more conservative approach is advisable in Child C patients with acute conditions and definitive surgery is recommended as an elective procedure after the liver function has improved.

KEY WORDS: Cirrhosis cholelithiasis surgery child's classification

### INTRODUCTION

Biliary tract surgery for gallstone disease in cirrhotic patients is reported to be a formidable undertaking because of operative blood loss from associated portal hypertension, increased rate of postoperative sepsis and subsequent liver failure[1–5]. Many authors recommend a conservative policy and suggest that surgery is only indicated for life-threatening complications such as empyema of the gallbladder, perforation and acute cholangitis[2–5]. Others, however, suggest that in mild cirrhotic patients elective cholecystectomy is a relatively safe procedure [6–8].

Due to the incidence of hepatitis-B in Taiwan the prelevance of post-hepatitic and post-necrotic liver cirrhosis is high[9], and we often encounter cirrhotic patients with symptomatic gallstone disease.

### MATERIALS AND METHODS

Between January 1983 and March 1988 112 patients with liver cirrhosis and gallstone disease were admitted, patients with malignant disease were excluded from this review. In 62 overtly cirrhotic patients liver cirrhosis could be diagnosed by past history, presence of portal hypertension, splenomegaly, laboratory findings and imaging studies. In the other 50 patients with occult cirrhosis the diagnosis was not made until laparotomy was performed. 96 patients had a liver biopsy, 92 had

*Address for correspondence*: Wu CC. 160, sec. 3., Chung-Kang Rd. Department of Surgery Taichung Veterans General Hospital Taichung, Taiwan, 40705, R.O.C.

post-necrotic cirrhosis, in 3 it was alcoholic and in 1 it was biliary in aetiology. No histological confirmation of cirrhosis or information about the aetiology was available for the remaining 16 patients.

Eighty seven out of 112 patients underwent definitive surgical procedures such as cholecystectomy or extraction of gallstones. Biliary surgery was considered to be an emergency procedure in 27 patients because of acute cholecystitis or acute obstructive cholangitis, which was unresponsive to aggressive medical treatment. The other 60 patients, including 8 asymptomatic with occult cirrhosis, had elective biliary tract operations. Definitive surgery was carried out in symptomatic cirrhotic patients with a favourable liver function as either an elective or emergency procedure. Patients without symptoms or with mild symptoms which responded to medical treatment did not go to surgery if liver function was poor. During the same period 1142 other patients received definitive surgical procedures for their gallstones.

The procedures carried out on the 87 patients having definitive treatment were cholecystectomy in 45, cholecystectomy and choledocholithotomy in 35, Roux-en-Y hepaticojejunostomy for reflux ascending cholangitis in 1, choldocholithotomy in 7 patients who had previously had a cholecystectomy. Palliative procedures were carried out in the other 25 overt cirrhotic patients with poor liver function, 20 of whom presented with acute biliary symptoms. Cholecystolithotomy was carried out in 5 patients with acute cholecystitis and of the 15 patients presenting with acute obstructive cholangitis, transhepatic biliary drainage[10] was performed in 4 and endoscopic papillotomy and drainage in 11 patients[10]. The other 5 patients with very poor liver function did not receive any treatment for their gallstones as they were asymptomatic.

All patients were divided into three grades according to Child's prognostic criteria for liver cirrhosis[11]. The points assigned to the various criteria are shown in Table 1. A score of 5–7 was graded as A, 9–10 as B and a score of more than 11 grade C. The laboratory data, operative blood loss and total blood transfusion, the nature of the operation and outcome were analysed. Up to March 1990 all patients had been followed up for at least 2 years or until their death (range 24–102 months, average 52.8 months). The statistical analysis was by one-way Anova test or Mann-Whitney's U test.

**Table 1** Child's prognostic criteria for liver cirrhosis

| variables value | 1 | 2 | 3 |
|---|---|---|---|
| serum albumin (g%) | > 3.5 | 3.0–3.5 | < 3 |
| serum bilirubin (mg%) | < 2 | 2–3 | > 3 |
| ascites | none | controlled | poorly controlled |
| encephalopathy | none | minimal | coma |
| nutrition | good | fair | emaciated |

### RESULTS

The location of the stones in the biliary system of patients undergoing definitive surgical procedures is shown in Table 2. Thirty four patients (39.1%) had stones in the bile duct. Of the 87 patients operated on definitively 2 were Child's B and 18 were Child's C. All patients who did not receive treatment for their biliary tract disease were Child's C. Details of patients undergoing definitive surgical procedures are shown in Table 3. There was no significant difference between the three grades for age, sex or proportion with overt cirrhosis. However, the incidence of emergency surgery, operative blood loss and total blood requirement and postoperative hospital stay were significantly less in the Child's group A than in the other grades. Postoperative complications occurred in 13 patients (14.3%) of the definitive surgery group. These are shown in Table 4. Four (4.6%) died within 28 days of the operation. These were all in the Child's C group and had emergency surgery for bile duct stone and acute suppurative cholangitis. Death was due to multiple organ failure. Of the patients undergoing palliative treatment 4 patients (20%) died within 28 days. One patient died of hepatic failure 5 days after cholecystolithotomy, 2 patients died from severe sepsis in spite of percutaneous biliary drainage for acute cholangitis. Bleeding occurred in 1 patient during endoscopic papillotomy and in spite of urgent surgery to remove the bile duct stone and control the bleeding, death occurred from progressive hepatic failure on the 28th postoperative day. Of the 83 immediate survivors after definitive surgery, 2 died from massive variceal bleeding at 6 and 37 months. Two died of hepatocellular carcinoma at 46 and 74 months and 1 died of hepatic failure after 14 months. Two patients are alive following subsequent peritone-

**Table 2** The location of the stones during definitive biliary surgery

| | |
|---|---|
| GB | 53 |
| GB + CBD | 11 |
| CBD + IHD | 8 |
| GB + CBD + IHD | 5 |
| IHD | 5 |
| CBD | 3 |
| GB + IHD | 2 |
| | 87 |

Note: GB: gallbladder
CBD: common bile duct
IHD: intrahepatic duct

**Table 3** Preoperative data and operative result by Child's grading in definitive surgically treated patients

| | A (n=39) | B (n=14) | C (n=34) | Significance | |
|---|---|---|---|---|---|
| Sex | male 31 | male 7 | male 26 | NS | |
| Age (range) | 57.6 (31–76) | 57.1(37–71) | 62.1 (33–82) | NS | |
| Emergent operations | 7 | 6 | 14 | A vs B<br>Avs C | p<05 |
| Overt cirrhosis | 12 | 8 | 17 | NS | |
| Postoperative hospital stay average (range) days | 10.3(7–22) | 17.6(8–44) | 17.5(3–55) | NS | |
| Operative blood loss average (range) | 289.3ml<br>(0–2000) | 1088.9ml<br>(50–3000) | 778.2ml<br>(50–3500) | A vs B<br>A vs C | p<0.05 |
| Blood transfusion average (range) | 102.4ml<br>(0–2000) | 732.1ml<br>(0–2500) | 589.7m;<br>(0–3500) | A vs B<br>A vs C | p<0.05 |

ovenous shunting at 16 and 82 months after biliary surgery. Seventeen survivors with intrahepatic stones required postoperative choledochoscopy on several occasions during the 6 months after their surgery to remove stones.

Two patients were found to have recurrent gallbladder stones 73 and 89 months after cholecystolithotomy. One of these patients required a further cholecystolithotomy. Cholangioscopic choledocholithotomy was used to remove bile duct stones in 12 patients who recovered from acute cholangitis. Eleven patients (55%) of those who survived after palliative procedures died of complications of their liver disease within 84 months of the stone removal. Only two patients who did not receive any treatment survived to be discharged from hospital. They both died of liver failure within 16 months.

### DISCUSSION

In 1980, McSherry *et al.* found that liver cirrhosis was one of the major causes of death in benign biliary tract surgery[12]. The following year, Schwartz[1] reported that biliary tract surgery in cirrhotic patients was dangerous due to excessive peri-operative bleeding caused by portal hypertension, which increased the incidence of postoperative sepsis and multiple organ failure. A rather conservative approach was therefore taken when in cirrhotic patients with gallstone disease. Aranha *et al.*[2,3] and Castaing *et al.*[5] hold that cholecystolithotomy is safer than cholecystectomy for patients with gallbladder stones and liver cirrhosis. Bornman *et al.*[13] recommended subtotal cholecystectomy for difficult gallbladders in patients with portal hypertension to minimize the operative blood loss. Cholecystectomy and common duct exploration in cirrhotic patients are held to be suitable only for life-threatening complications of biliary tract stones[2,3,14]. However, elective cholecystectomy in mild cirrhotic patients is considered to be safe, the morbidity between cirrhotic and non-cirrhotic patients is not different. Bloch *et al.* suggest that elective surgical interventions are warranted in cirrhotic patients with Childs A and B grade for symptomatic cholelithiasis[6].

**Table 4** Postoperative complications in 13 patients who underwent definitive biliary surgery (4 patients had more than 1 complication)

| | |
|---|---|
| 1. Bile leakage | 3 |
| 2. Wound infection | 4 |
| 3. Upper gastrointestinal bleeding | 3 |
| 4. Ascites leakage | 4 |
| 5. Acute renal failure | 1 |
| 6. Wound dehiscence | 2 |
| 7. Atelectasis | 1 |
| 8. Hepatic failure | 2 |

Owing to the high prevalence of hepatitis B in Taiwan[9], the incidence of postnecrotic liver cirrhosis is also high with less that 10% not being of postnecrotic origin. Because of our strict patient selection, the overall mortality in this series was not high, with no operative death in Child's A and B groups, but still 4 patients with Child's C grade and acute cholangitis died. Child's B and C group patients had more operative bleeding and blood requirement than Child's A patients and their hospitalization was also longer. In the report of Ma *et al.*[8] more operative blood loss and longer hospitalization were observed in elective cholecystectomy in cirrhotic rather than in non-cirrhotic patients. From our results, we find that in Child's A patients, the coagulation function was better than in the other two groups, the chance of portal hypertension was lower, and operative bleeding was therefore less and their postoperative recovery more rapid. So we suggest that the Child's A

cirrhotic patients can be regarded as non-cirrhotic in biliary tract surgery.

Stones in the bile duct in cirrhotic patients may complicate the disease, especially when present with obstructive jaundice, which causes marked deteriorization of liver function. Chen *et al.*[14] and Shrinlk *et al.*[15] report that the mortality is higher in cirrhotic patients if their bile duct is opened. Because of the high prevalence of hepatolithiasis in Taiwan[16], we frequently encounter patients with stones in the bile duct which causes cholangitis and ideally should be treated with bile duct exploration. These patients are always emergency and have higher risks as suggested by Castaing[5]. In these conditions, it is essential to distinguish the jaundice caused by cirrhosis per se from that of bile duct obstruction, and avoid unnecessary bile duct exploration. Preoperative biliary tract imaging is therefore more important for Child's C cirrhotic patients. Aranha *et al.*[3] recommend endoscopic retrograde cholangiogram or percutaneous transhepatic cholangiogram for cirrhotic patients with jaundice. The eight patients who had surgery in that series had surgery for acute cholecystitis of which 5 were cholecystostomy and three cholecystectomy. There were 13 patients who had jaundice and cirrhosis. In them, 8 patients had endoscopic retrograde cholangiography, 4 had transhepatic cholangiography, and 1 had cholecystectomy with common bile duct exploration. For Child's C group patients, the palliative approach is probably more practical. After 1987, we treated severe cirrhotic patients with bile duct stone by endoscopic biliary drainage and then removal of stones. Although one death occurred due to technical complications, the other patients survived the acute cholangitic episode. Endoscopic drainage can not only can make the biliary tree, but also drain the infected bile. Percutaneous transhepatic cholangioscopy was also reported to be effective in treating retained bile duct stones[17]. However, for some cirrhotic patients with ascites, we do not think that it is suitable.

Although cholecystolithotomy is suggested to be safer in cirrhotic patients with gallstone and acute cholecystitis[3] we think that it is true only for Child's C patients. Definitive biliary surgery can be carried out at any time in patients with favorable liver function. We do not recommend a conservative policy for cirrhotic patients with good liver function, because of the high chance of recurrent disease after palliative procedures[18]

In conclusion, definitive biliary surgery was safe in Child's A and B cirrhotic patients . A palliative policy suggested for Child's C patients with acute conditions, and definitive procedures could be considered when liver function improves.

### REFERENCES

1. Schwartz, S. I. (1981). Biliary tract surgery and cirrhosis: a critical combination. *Surgery,* **90**, 577–783.
2. Aranha, GV., Semtag, S. J., Greenlee, HB. (1982). Cholecystectomy in cirrhotic patients: a formidable operation. *American Journal of Surgery,* **143**, 55–60.
3. Aranha G. V, Kruss D, Greenlee HB (1988). Therapeutic options for biliary tract disease in advanced cirrhosis. *American Journal of Surgery,* **155**, 374–377.
4. Cryer, H. M., Howard, D. A., Garrison, R. N. (1985). Liver cirrhosis and biliary tract surgery: assessment of risk. *Southern Medical Journal,* **78**, 138–141.
5. Castaing, D., Honssin, D., Lemoine, J., Bismuth, H. (1983). Surgical management of gallstones in cirrhotic patients. *American Journal of Surgery,* **146**, 310–313.
6. Bloch, R. S., Allaben, R. D., Walt, A. J. (1985). Cholecystectomy in patients with cirrhosis– a surgical challenge. *Archives of Surgery,* **120**, 669–672.
7. Kogut, K., Aragoni, T., Ackerman, N. B. (1985). Cholecystectomy in patients with mild cirrhosis– a more favorable situation. *Archives of Surgery,* **120**, 1310–1311.
8. Ma, S., Liu, M., Lui, Y. W., Peng, F. K., Mok, K. T., Lee, H. C. *et al.* (1988). Cholecystectomy in cirrhotic patients. *Journal of Surgical Association ROC.,* **21**, 1–6.
9. Chen, D. S., Sung, J. L., Lai, M. Y. (1978). A sero-epidemiologic study of hepatitis B virus infection of Taiwan. *Journal of Formosan Medical Association,* **77**, 908–918.
10. Stanley, J., Gobien, R. P., Cunningham, J., Andriole, J. (1986) Biliary decompression. An institutional comparision of percutaneous and endoscopic methods. *Radiology,* **158**, 195–197.
11. Christensen, E., Schlichting, P., Fauerholdt, L., Glund, C., Andersen, P. K., Juhl. E., *et al.* (1984). Prognostic value of Child-Turcotte criteria in medically treated cirrhosis. *Hepatology,* **4**, 430–435.
12. McSherrry, C. K., Glenn, F. (1980). The incidence and causes of death following surgery for non-malignant biliary tract disease. *Annals of Surgery,* **191**, 271–275.
13. Bornman, P. C., Terblanche, J. (1985). Subtotal cholecystectomy for the difficult gallbladder in portal hypertension and cholecystitis. *Surgery,* **98**, 1–6.
14. Chen, M. F., Jan, Y. Y., Chen, F. F., Wang, J. S., Chen, C. W., Jeng, L. B., *et al.* (1982). Fatal complication in biliary surgery for cirrhotic patients. *Journal of Formosan Medical Association,* **82**, 977–981.
15. Shrinlk, K. R., Burk, R. R., Brown, M., Levine, B. A (1987). Improving survival in patients with cirrhosis undergoing major abdominal operations. *Archives of Surgery,* **122**, 271–273.
16. Nakama, F., Koga, A (1984). Hepatolithiasis present status. *World Journal of Surgery,* **8**, 9–14.
17. Chen, M. F., Jan, Y. Y., Lee, T. Y. (1987). Percutaneous transhepatic cholangioscopy. *British Journal of Surgery,* **74**. 728–730.
18. Dempsey. D. T., Rosato, E. F. (1985). Surgical management of cholelithiasis in gallstones. Edited by Cohen S, Soloway RD, pp. 191–213. New York, Churchill Living Stone.

